# Effects of daridorexant on attentional performance in adults with Attention-Deficit/Hyperactivity Disorder (ADHD)

**DOI:** 10.1192/j.eurpsy.2026.11992

**Published:** 2026-07-31

**Authors:** G. Grassi, E. Scillitani, C. Moradei, C. Cecchelli

**Affiliations:** Brain Center Firenze, Florence, Italy

## Abstract

**Introduction:**

Sleep disorders, particularly insomnia, are highly prevalent in adults with ADHD, worsening core symptoms and impairing cognitive functioning. Despite their clinical relevance, evidence-based treatments for insomnia in this population remain limited, with most studies focusing on pediatric samples. Dual orexin receptor antagonists, such as daridorexant, represent a novel therapeutic option but have not yet been investigated in adults with ADHD.

**Objectives:**

The primary aim of the present study was to evaluate the effects of daridorexant on sleep outcomes in adults with Attention-Deficit/Hyperactivity Disorder (ADHD) and comorbid insomnia disorder. Within this framework, the present work reports a planned sub-analysis specifically focused on cognitive outcomes. In particular, we aimed to investigate whether improvements in sleep parameters following daridorexant treatment would be associated with measurable effects on attentional performance, as assessed by the Conners’ Continuous Performance Test 3 (CPT-3). This sub-analysis was designed to explore the potential impact of sleep enhancement on vigilance, attention and impulsivity, domains that are typically impaired in adults with ADHD.

**Methods:**

In a naturalistic, open-label study, 24 adults with ADHD and comorbid insomnia disorder received daridorexant 50 mg daily for one month. Assessments included subjective sleep measures (Insomnia Severity Index, Sleep Condition Indicator), objective sleep parameters (actigraphy), and cognitive performance evaluated with the Conners’ Continuous Performance Test 3 (CPT-3).

**Results:**

Daridorexant was associated with significant improvements in both subjective and objective sleep quality, including increased total sleep time and sleep efficiency and reduced sleep onset latency. In the full sample of 24 participants, two CPT-3 indices improved significantly: Hit Reaction Time Standard Deviation (HRT SD), indicating more stable responses and better vigilance, and Hit Reaction Time Block Change (HRT Block Change), reflecting enhanced sustained attention. Conversely, Hit Reaction Time Inter-Stimulus Interval Change (HRT ISI Change) worsened, suggesting difficulties in maintaining vigilance under low-stimulation conditions. However, when excluding the three participants with no benefit or worsening of sleep, this negative effect was no longer observed, and further gains emerged, including improvements in Detectability—indicating better discrimination between target and non-target stimuli (selective attention)—and in Commissions, reflecting reduced impulsivity. Notably, improvements in cognitive indices correlated with subjective improvements in insomnia symptoms.

**Conclusions:**

In adults with ADHD and comorbid insomnia, daridorexant improved both sleep and cognitive measures, with cognitive gains closely linked to sleep improvement, although further studies are required to confirm these findings.

**Disclosure of Interest:**

None Declared

